# We know what we want, it’s just not there

**DOI:** 10.1017/cts.2021.882

**Published:** 2021-12-16

**Authors:** Robert E. Freundlich, Christopher J. Lindsell

**Affiliations:** 1 Department of Anesthesiology, Vanderbilt University Medical Center, Nashville, TN, USA; 2 Department of Biomedical Informatics, Vanderbilt University Medical Center, Nashville, TN, USA; 3 Department of Biostatistics, Vanderbilt University Medical Center, Nashville, TN, USA

In today’s academic health centers, our research, teaching, and clinical missions are thirsty for data. Indeed, who among us could do our jobs without data? Mostly, we’re pretty sure we know what we need. It is right there in the electronic health record (EHR). We can open a patient’s chart, swiftly navigate to the data point, and declare our problem solved – we know there are data. Most commonly, however, when we try to pull the data, we encounter significant hurdles. Why the disconnect?

Perhaps on our computer screen, we were pointing to a patient’s non-invasive systolic blood pressure – flow sheet row 483797983721. What is not clear at first sight is that this flow sheet row is stored in three separate relational tables, part of a relational database with tens of thousands of such tables. These tables are automatically extracted, transformed, and loaded in a series of scheduled jobs, where they are disseminated to no less than four new databases, often in a cascading fashion. While flow sheet row 483797983721 looked perfect on the screen, the subsequent databases may have significantly modified its appearance or availability. The systolic and diastolic blood pressure may be merged into one cell, separated by a dash – easy enough to fix with a few lines of code. Or the systolic blood pressure from a non-invasive blood pressure cuff may be merged with invasive blood pressure measurements from an arterial line – impossible to separate. Even worse, systemic arterial blood pressure measurements may be mixed in with other pressure measurements, such as pulmonary arterial blood pressures, due to incorrect or inaccurate mapping of data (in fairness, they do all contain the string “arterial blood pressure”). The more proximal databases are unavailable to researchers by design. As a result, our experience is that data scientists and researchers using “EHR data” are using data that are many steps removed from the truth. We are reminded of the game “telephone.”

Trying to convey the complexity of data pathways behind a modern, enterprise EHR, with a cascade of hierarchical data sources, is challenging. Even the most attentive data consumer struggles with understanding the provenance of their dataset. Perhaps it is time to rethink the “data basis” for our academic health centers. Today, each data source, with the limitations and dependencies imposed on it, is imperfect. Yet, each has a role in the broader enterprise and was designed for a specific purpose. Our experience is that, by not including key data stakeholders in the inception and design of critical databases, we typically achieve our intended purpose in a manner that limits other appropriate uses of the data. Addressing this concern will require a drastic overhaul of existing workflows and priorities and increased data literacy among users to understand which data source is appropriate for what purpose. Building a culture of data literacy amongst stakeholders will be challenging, unless it is relentlessly driven by leaders willing to take the responsibility and ownership to tackle this critical issue. We believe that this is achievable but, similar to other examples of culture and paradigm shift, it will require significant ongoing investment and human capital. As such, buy-in from institutional leadership will be essential.

While involving stakeholders throughout the design and implementation process would be ideal, we recognize that this may not always be feasible – or even achievable in the short term. As an alternative, we propose more intelligent use of existing data sources. Data pathways and data needs will vary by project. An agile, adaptable platform, able to navigate the complex offering of potential data sources to meet individual needs, may offer significant benefits. Rather than relying solely on a single data source, such as an electronic data warehouse, a more flexible and diverse menu of options is needed. A mix of structured and unstructured data should be made available, with varying levels of artifact reduction and processing offered. Critically, institutional leaders should push for increased access to more “proximal” sources of data for researchers and data scientists so that they too may access the least-processed data sources, where appropriate.

Given our diverse needs, ubiquitous reliance on data, current state of data resources, and the momentous growth we are experiencing, data scientists and data stakeholders owe it to one another to work reciprocally to address the data pipeline. Institutional leadership should be actively engaged in the challenge, with clear responsibilities and ownership outlined. As a result of improved data literacy, with a focus on understanding the data pipelines, we expect it will become possible to point to a data point on a screen in the EHR and easily reproduce it in an operational, clinical, or research dataset. Without that, the meaning and information content of our data are in doubt. The explosion of cheap data storage and massive computational power makes this the perfect time for a seismic shift in our approach to data. While data warehouses and repositories serve as a vital data resource, if we do not tackle the problem of understanding how a data element from a database ties back to what is observed on the computer screen, the framework for biomedical discovery using the EHR could be as flawed as a game of telephone.

